# The incidence of complications of central venous catheters at an intensive care unit

**DOI:** 10.4103/1817-1737.32232

**Published:** 2007

**Authors:** A. H. Akmal, M. Hasan, A. Mariam

**Affiliations:** *Consultant Intensivist, Department of Intensive Care Unit, Salmaniya Medical Complex, Manama, Bahrain*

**Keywords:** Central venous catheter, complications, infections, intensive care unit

## Abstract

Central venous catheter (CVC) placement in the intensive care unit (ICU) is a common practice and is being increasingly used also in general wards. Its use is associated with both mechanical and infectious complications.

**OBJECTIVE:**

To determine the infectious and mechanical complication rate of central venous catheterization in an ICU.

**DESIGN:**

A retrospective study about complications of 1319 central venous catheter placements.

**SETTING:**

An 11-bed adult medical, surgical, neuro-trauma ICU at salmaniya medical complex, *Bahrain*.

**MATERIALS AND METHODS:**

This was a retrospective review of all central venous catheter inserted over 4 year's period from October 2002 to December 2006.

**RESULTS:**

There were 12 mechanical complications and 128 infectious complications total of 1319 CVCs placed.

**CONCLUSIONS:**

The CVC can be performed safely in an ICU if done by a competent physician with all aseptic precautions.

Central venous catheters (CVCs) are widely used in critically ill patients throughout the world. They permit hemodynamic monitoring and allow reliable access for the administration of fluids, blood products, medications and total parenteral nutrition (TPN).[1]

The use of CVC is routine in critically ill patients, in fact, in the European Prevalence of Infection in Intensive Care (EPIC) study, 78% of the patients had some form of CVC inserted[2] Central venous catheterization may cause different complications including infection, hemorrhage and thrombosis. Mortality in catheter-related infection is the main reason for the widespread interest in CVC.[3] The question as to which venous catheterization site is associated with the highest risk of infection, remains controversial. Most catheter-associated infections are caused by Gram-positive (GP) organisms, in particular coagulase-negative staphylococci, though Gram-negative (GN) organisms still account for up to 28% of these infections.[4] Catheter-related bloodstream infections (CRBIs) are a cause of significant morbidity and mortality in intensive care unit (ICU) patients. Development of CRBIs may occur by several mechanisms. Our objective is to study the infectious and mechanical complication rates of percutaneously placed femoral and nonfemoral central venous catheters in critically ill adult patients.

## Materials and Methods

We studied the infectious and mechanical complication rates of percutaneously placed femoral and non-femoral central venous catheters in critically ill adult patients in a retrospective manner in an adult medical, surgical, neuro-surgical ICU. This study was conducted over a 4 year period from October 2002 to December 2006, The monthly admission to this ICU ranging from 45–50 cases. The average acute physiology and chronic health evaluation II (APACHE II) was 22 with a mortality of 17%. we used all multi-lumen catheters for central venous lines.

**Sterilization:** When inserting a catheter, we used maximal sterile-barrier precautions including a mask, a cap, a sterile gown, sterile gloves and a large sterile drape. Povidone-iodine based solutions were used to prepare the skin before insertion of the catheter.

**Culture for suspected CRI:** Once catheter-related infections were suspected, all catheter sites were examined carefully. If there was any purulence or erythemas, an exit-site infection was considered likely and the catheter removed. Two samples of blood were drawn from peripheral sites for culture to evaluate the possibility of bacteremia. This was done because it is difficult to determine whether a positive culture of blood from a CVC indicates contamination of the hub, catheter colonization or a catheter-related bloodstream infection. Due to this difficulty in establishing the possibility of bacteremia from a CVC, besides the peripheral blood sampling, all suspected CRI catheters were removed and the tips sent for culture along with the blood.

## Result

A total number of 1319 CVCs were inserted using Seldinger technique. 579 CVCs (43.8%) were inserted through the femoral vein (FV), 464 CVCs (35.1%) inserted through the internal jugular vein (IJV) and 276 (20.9%) through the subclavian vein (SV). Out of these 1319 catherizations, 128 (9.7%) became infectious complications. We found that the FV was more susceptible to infection with an incidence of 88/128 (15%) whereas IJV infection was seen in 20/464 (4.3%) patients. FV infection was caused mainly by *Staphylococci epidermidis* (staph-epi; 34%), *Staphylococcus aureus* (staph. aur.; 26%). Forty-two percent of the 1319 CVCs placed during this interval were femoral. Noninfectious complications were recognized in 1.5% of femoral catheters and 0.5% of nonfemoral catheters. Mechanical complications were manifested as hematomas of which 2/464 (0.43%) formed in the neck in IJV catheters. However, these hematomas subsided without any intervention or blood transfusion. Two patients out of 200 (0.72%) developed pneumothorax during subclavian vein catheter insertion. Both patients required chest tubes which were removed after five days. Nine out of 579 patients (1.5%) who had had CVCs inserted through the FV developed hematomas out of which one patient developed ilio-femoral deep vein thrombosis and infection at the site of insertion and expired. All procedures were performed by a well experienced intensivist or by an experienced senior resident.

A comparison among three central venous line sites based on culture results showed that the infection rate was 9.7% (*n=* 128/1319). For the IJV site, 234 catheter tips has been sent for culture. Twenty of these catheter tips were found to be colonized by bacteria thus bringing the infection rate at the IJV site to 4.3%. Central lines inserted in the subclavian site showed a 10% infection rate as 28 catheters tips out of 276 was infected. The femoral site showed the highest infection rate (15.1%) as 88 out of 579 catheters were found infected. There was occurrence of total 12 mechanical complications out of the 1319 CVCs inserted (0.98%).

## Discussion

CVCs are an important tool in the operation room and ICU. The use of CVCs is associated with both mechanical and infectious complications.[5]

Out of 1319 CVCs inserted, 619 tips of the catheters were sent for culture and sensitivity. One hundred and twenty eight of these catheter tips were positive fr bacterial cultures for different organisms. Hence, all infected CVCs were removed and the patients were treated with appropriate antibiotics.

CVC-related systemic infections were found in 15% of patients with femoral catheters, 10% with subclavian catheters and 4.3% with internal jugular catheters. Catheters inserted during emergency situations were exchanged within first 24 hours. All procedures being performed by a qualified intensivist or a competent senior resident under strict aseptic precautions. Cultures were performed on the tips of the catheters using endothelial brushing and maki roll techniques.

Catheter-related sepsis is a well known complication in critically ill patients receiving total parenteral nutrition.[6] Micro-organisms may travel from the skin puncture wound along the external surface of the catheter or from the hub through the lumen of the catheter, to be shed into the circulation causing bacteraemia and sepsis. The incidence of sepsis is said to be about three times greater with multiple-lumen catheters than with single-lumen catheters.[6]

Skin organisms colonizing the distal intravascular tip of the catheter ultimately cause bloodstream infection.[7] Hub contamination is more common in long-term catheters because such catheters often have to be intercepted and manipulated.[7]

Organisms are usually introduced into the hub from the hands of medical personnel. From this contaminated hub, the organisms migrate along the internal surface of the catheter, where they can cause a bloodstream infection.[8] Fever and signs of sepsis, such as chills, rigors, hypotension and hyperventilation should always be considered as CRI when there is no other identifiable source of infection is present. But clinical findings are unreliable for establishing a diagnosis of CRI.[8]

Catheter-associated infections can be considered local or systemic. Local phenomena include simple colonization or true infection that may involve the exit site or tunnel. Local inflammatory signs at the catheter's portal of entry or tunnel have a highly predictive value for infection but its absence has a very poor negative value.[9]

The skin insertion site and the catheter hub are by far the two most important sources; approximately 65% of CRI originate from the skin flora, 30% from the contaminated hub and 5% from other pathways.[10]

Quantitative blood culturing techniques have been developed as alternatives for the diagnosis of catheter-related bloodstream infection in patients for whom catheter removal is undesirable because of limited vascular access.[11] The practice of routinely changing catheters in a predefined time period to reduce the risk of CRI is referred to as “scheduled” replacement. There is no support from the literature that catheter replacement at scheduled time intervals will reduce the CRI rates.[12] The risk of complication during the insertion or exchange of CVCs has been well documented. The majority of complications involve mechanical problems associated with insertion. Although cardiac arrhythmia has been acknowledged as a possible complication, its incidence has never been quantified.[13]

In critically ill patients, barotrauma and puncture of an incompressible artery are probably the most common mechanical complications and can be life-threatening. The rate of mechanical complications has ranged from 0–12%, according to the experience of the operator and the definition of complications;[14] Mechanical complications include arterial puncture, pneumothorax, mediastinal haematoma, haemothorax and injury to adjacent nerves. The recent introduction of more flexible catheters and of the J guide-wire insertion method has decreased the rate of severe mechanical complications. Although fatal complications still occur,[15] in our study, the mechanical complication rate was 0.9%.

In the meta-analysis by Ruesch and coworkers,[16] arterial punctures were significantly more common with the jugular than with the subclavian approach (six trials, 2010 CVCs; 3% *vs* 0.5%; relative risk (RR) 4.7, 95% confidence interval (CI) 2.05–10.77). However, bleeding from a punctured internal carotid artery can usually be controlled by manual compression. A hematoma may occur, though, particularly when a dilator or pulmonary artery catheter is inserted in a patient with haemostasis disorders. A large hematoma may produce rare but serious complications including airway obstruction, retrograde aortic dissection, arteriovenous fistula or cerebrovascular events in patients with occlusive atheromatous disease of the carotid artery.[16]

Most studies have demonstrated that the use of prophylactic antibiotics is associated with reduction in the rate of catheter-related bloodstream infections.[17] However, this use of antibiotics is discouraged because of the concern that it will encourage the emergence of antibiotic-resistant organisms.[18] As with most medical procedures, the level of experience of the physician reduces the risk of complications.[19] Insertion of a catheter by a physician who has performed 50 or more catheterizations is half as likely to result in a mechanical complication as an insertion by a physician who has performed fewer than 50 catheterizations.[19] The incidence of mechanical complications after three or more insertion attempts is six times the rate after one attempt.[20] Hence, if a physician is unable to insert a catheter after three attempts, he or she should seek help rather than continue to attempt the procedure.

## Conclusion

The choice of the best central venous access for a particular patient is based on the rate and the severity of failures and complications. Based on our experience, internal jugular access is associated with a low rate of severe mechanical and infectious complications in the intensive care unit as compared with subclavian and femoral access. We conclude as per our experience that CVCs play an important role in treating critically ill patients in an adult ICU but may cause serious multiple mechanical and infectious complications if proper precautions are not taken.
